# Supplementary material for: A follow-up study (2007–2018) on American Tegumentary Leishmaniasis in the municipality of Caratinga, Minas Gerais State, Brazil: Spatial analyses and sand fly collection
Source: PLoS Negl Trop Dis. 2021 May 18;15(5):e0009429. doi: 10.1371/journal.pntd.0009429 (PMC8162678; doi:10.1371/journal.pntd.0009429)
Supplement: Attachment — Submitted filename: Answer to reviewers R1.doc [file pntd.0009429.s001.doc]

**Reviewer #1:** p6, line 188: If the relative risks were already estimated using Monte Carlo simulation, why was an OLS model used? What advantage does this offer over the Bayesian spatial model which has already been performed? (The explanation on p12 lines 360 to 362 isn’t convincing. A Bayesian model could just as easily do this).

**This is interesting point raised by the reviewer, however the Monte Carlo simulation was used to identify the significance of the clusters, as mentioned in the manuscript: “The significance test of the identified clusters was based on the comparison of a null distribution obtained by Monte Carlo simulation” in the item “Scanning analysis of space-time clusters”. On the other hand, OLS model was used for spatial regression, aiming to identify behavioral factors for each census tract sector that could explain differences in the occurrence of leishmaniasis. Our results presented in table 4, suggests that 56% of leishmaniasis cases can be explained by census tracts with family income up to ½ minimum wage and irregular waste disposal on land.**

**The Bayesian model was used with the intention of reducing random fluctuations that may occur due to areas with small populations and few cases may imply variations in incidence rates. These were explained in the manuscript “and the smoothing was performed to reduce random fluctuation. This facilitates the subsequent analysis of spatial data, since areas with small populations and few cases may imply great variation in their rates” in the item “Bayesian analysis.”**

Figure 4: It would be useful having a map inset for the urban area of Caratinga in each plot (similar to Figure 7) to improve clarity.

**Figure 4 has been changed as requested.**

Page 10 to 14: The Discussion should be limited to a discussion of the results of the study, limitations of the methods, caveats, and suggestions for future research. There is a lot of content here that is a reiteration of the introduction or new background (e.g. lines 271-280, lines 377-395, lines 397-405), introduction of new data/methods (e.g. lines 305), results rather than a discussion of them (e.g. lines 286-293, lines 395-396), and otherwise superfluous content like explanation of methods (e.g. lines 308, 326).

**We agree with the reviewer and all excessive information was shortened and/or removed (now lines 268-294).**

Additionally, the purpose of the two hypotheses mentioned on line 293 is unclear. Is this speculation or a suggestion for future research to address?

**We agree with the reviewer that the choice of the word was not good, and we replaced it for reasons. This is more a discussion rather than a suggestion for future studies. What we implied there is that those two possibilities made us to look for infected vectors in the urban area to pursue the possibility of ATL urbanization/transmission, an important contribution of our manuscript.**

p4, lines 120 to 122: I suggest a rewording of this sentence to be clearer, e.g. “Depending on the city, peridomiciliary/urban transmission and/or sylvatic transmission may be observed.”

**The sentence as corrected as requested (now lines 117-118): Depending on the city, peridomiciliary/urban transmission and/or sylvatic transmission may be observed.**

p5, lines 143 to 151: I don’t know why the study area and statement on ethics approval have been lumped together. I think ethics approval would be better suited elsewhere.

**Following the reviewer´s suggestion, a topic was created where the ethical information was included as described below (now lines 140-142):**

p5, line 151: This last sentence is unclear. Does this mean all data involved was de-identified? This seems to be stated on line 155 already, so I suggest omitting it here.

**The information was omitted as requested.**

p5, lines 157 to 158: Why was it necessary to categorise patients into three-year intervals to analyse their spatial distribution? Is it because there are too few cases for each year individually, or some other reason? It would be helpful to add this reasoning.

**Since Leishmaniasis has a periodicity grouping in three-years interval will help to minimize the effects of high or low incidence that is common especially considering the size of the area studied. In this context, the combined incidence helps to better understand the temporal and spatial occurrence of cases. We are familiar with this methodology that we already used it a previous paper (Cardoso et al., 2019) [ref. 35]. This information was included as requested (now lines 163-164).**

p5, lines 160 to 162: The terms “…empirical spatial smoothing” and Bayesian “spatial smoothing” don’t need to be capitalised.

**The corrections were included as requested.**

p5, lines 161 to 162:  I may be misunderstanding the term “accumulated” in this sentence, but the incidence rate should be estimated for each combination of area and demographic sector. However, only the demographic sectors are explicitly mentioned.

**The term accumulated refers to the incidence during the three-year period. We changed the sentence as follows (lines 172-173): After calculating the incidence rate, the accumulated smoothed incidence rate (three-year period) (bayesian spatial smoothing) was calculated for each demographic sector.**

p5, line 163: The term “queen” is out of place in the sentence. Moreover, it is not a very useful term when relating to non-lattice geographic units. I suggest describing the spatial weights matrix as “A first-order adjacency matrix”, as is common practice with this type of spatial data.

**The term "queen" was removed, and the sentence was re-written as suggested (line 167).**

p6, line 168: The first sentence here isn’t clear (the word “ellipse” by itself does not indicate what type of analysis/test is being performed).   I suggest deleting this sentence, and begin this section with “Directional distribution ellipses provide…”.

**The suggestion was included as requested (lines 172 - 173).**

p6, line 174: Similarly, the word “kernel” is not very descriptive. I suggest using “kernel density maps”.

**The suggestion was included as requested (line 177-178).**

p6, line 190 to 191: The reader should be familiar with the assumptions of OLS and it is not necessary to state this.

**That information was removed as requested.**

p6, lines 191 to 193: I suggest rephrasing this sentence to make it clearer these items were observed rather than estimated: “Population, garbage dumped…, sewage collection were included as explanatory variables”.

**The correction was included as follows (lines 192-194).**

p6, line 194: The meaning of “according to the variables” is unclear. Does this mean AIC is computed for model including all explanatory models?

**We agree with the reviewer and this term was removed.**

p7, line 202: The abbreviation “HP” is unclear.

**This abbreviation stands for Hoover Pugedo who manufactured the HP trap based on the CDC trap. For those working with sand flies collection it is very common used term that is why we prefer to keep the abbreviation HP and this information is in its respective reference (Pugedo et al., 2005) [46].**

p7, line 215: Use of parenthesis is inconsistent.

**The sentence was rewritten as follows (now lines 214-216):**

**Males (57.37%) represented the majority of the ATL cases followed by females (42.63%) (183 versus 136).**

p13, line 373: “analyzes” should be “analyses”. Similarly on p14, line 411.

**The correction was performed as requested (lines 318 and 353).**

p13, line 373: “this” should be “which”.

**The word was replaced as requested: (now line 319).**

Suggest rewording the beginning of this sentence to “The spatial analysis was carried out…”.

**The correction was included as requested (now line 355).**

p14, line 413: I suggest adding the word “district” in parenthesis to the second legend item, Patrocinio de Caratinga.

**As suggested, the word "district" was added between parentheses.**

**Reviewer #2:**

Title - include the title search for sand fly.

**Now the Title reads:** **A follow-up study (2007-2018) on American Tegumentary Leishmaniasis in the municipality of Caratinga, Minas Gerais State, Brazil: spatial analyses and sand fly collection.**

I suggest reducing the introduction of the abstract, which must be extremely brief, and place the reader on the subject covered in the study. Add the objectives and improve the description of the results, adding relevant discussions.

**We have rewritten the abstract according to reviewer’s suggestions and reduced the background information and included objectives (lines 17-23). We prefer not to include a discussion in the abstract to comply with PNTD guidelines, which requires mostly results and conclusion of the manuscript in the abstract.**

Pag. 1, Line 30 "Information was retrieved from the public health archives", all information? Specify which.

**The information was included in the abstract (lines 23-24).**

Pag. 1, Line 40: Improve the conclusion of the summary. What does the study bring again? "Based on our analyzes, the recent urbanization of ATL in Caratinga…" this information is already on the title.

**We agree with the reviewer that urbanization should be in the conclusion and not in the title, since this is the phenomenon that we want to report in the manuscript. We changed the abstract conclusion as follows (lines 32-36) and include this in the summary (lines 51-52).**

Introduction - Include sand fly research and leishmania research in the objectives.

**This information was added (lines 133-134).**

Pag 9, line 156: Add education level

**It was added here (line 160) and in the abstract (line 23).**

Study area - briefly characterize the municipality studied. PIB, climate, main economic production, so that whoever lives in another state or country, can understand the characteristic of the study area.

**This information was included (lines 148-152).**

Pag. 7, line 214: 319 cases notified in which period? Please specify.

**This information was included (lines 215-216).**

Pag. 8, line 242: I suggest removing "Confirming our previous observations", this statement is unnecessary in this paragraph.

**The sentence was removed.**

Pag. 8, line 245 - add this information to the methodology and describe only the result.

Pag. 8, line 250 - add this information to the methodology and describe only the result.

**Those information were sent to methodology, but to facilitate the readers understanding of the rational we kept methodology information to the minimum.**

Pag. 10, line 300 –Which places? It will be interesting to know some of them.

**Locations were added according to reviewer's request (line 285).**

Pag. 14, line 428 - The conclusion must be aligned with the objectives.

**We tried to reformulate the last paragraph according to reviewer´s suggestion (lines 371-378).**

Figure 4 - specify in the map legend and the figure legend if it is about the incidence and per 10,000 inhabitants. District names are unreadable in some places. I suggest removing it since the map specifying the districts with the names has already been presented previously.

**Figure 4 was changed as also requested by Reviewer #1 now Figures 4 and 5.**
